# The Effect of the 2015 Earthquake on the Bacterial Community Compositions in Water in Nepal

**DOI:** 10.3389/fmicb.2017.02380

**Published:** 2017-12-06

**Authors:** Sital Uprety, Pei-Ying Hong, Nora Sadik, Bipin Dangol, Rameswor Adhikari, Antarpreet Jutla, Joanna L. Shisler, Patrick Degnan, Thanh H. Nguyen

**Affiliations:** ^1^Department of Civil and Environmental Engineering, University of Illinois at Urbana Champaign, Urbana, IL, United States; ^2^Water Desalination and Reuse Center, Biological and Environmental Science and Engineering Division, King Abdullah University of Science and Technology, Thuwal, Saudi Arabia; ^3^Environment and Public Health Organization, Kathmandu, Nepal; ^4^Department of Civil and Environmental Engineering, West Virginia University, Morgantown, WV, United States; ^5^Department of Microbiology, University of Illinois at Urbana Champaign, Urbana, IL, United States

**Keywords:** microbial stability, perturbation, earthquake, opportunistic pathogens, Nepal

## Abstract

We conducted a study to examine the effect of seasonal variations and the disruptive effects of the 2015 Nepal earthquake on microbial communities associated with drinking water sources. We first characterized the microbial communities of water samples in two Nepali regions (Kathmandu and Jhapa) to understand the stability of microbial communities in water samples collected in 2014. We analyzed additional water samples from the same sources collected from May to August 2015, allowing the comparison of samples from dry-to-dry season and from dry-to-monsoon seasons. Emphasis was placed on microbes responsible for maintaining the geobiochemical characteristics of water (e.g., ammonia-oxidizing and nitrite-oxidizing bacteria and archaea and sulfate-reducing bacteria) and opportunistic pathogens often found in water (*Acinetobacter*). When examining samples from Jhapa, we identified that most geobiochemical microbe populations remained similar. When examining samples from Kathmandu, the abundance of microbial genera responsible for maintaining the geobiochemical characteristics of water increased immediately after the earthquake and decreased 8 months later (December 2015). In addition, microbial source tracking was used to monitor human fecal contamination and revealed deteriorated water quality in some specific sampling sites in Kathmandu post-earthquake. This study highlights a disruption of the environmental microbiome after an earthquake and the restoration of these microbial communities as a function of time and sanitation practices.

## Introduction

Safe drinking water requires that the microbial community remains stable to minimize the risk of pathogen propagation and release (Rittmann, 1984; Hu et al., 1999; Prest et al., 2016). The biological stability of drinking water during common water treatment processes and water distribution has been examined (Lautenschlager et al., 2013; Prest et al., 2014). However, the variation in microbial community as a result of sudden changes, such as a natural disaster, remain understudied. Earthquakes are one form of natural disaster that can negatively impact human health and have high economic and environmental costs. The April 2015 earthquakes in Nepal caused more than 5 billion USD in damage (Government of Nepal, 2015; Upadhya and Seikh, 2015). These earthquakes caused 8,959 fatalities, a significant increase in waterborne infection incidence (Simkhada et al., 2015), limited water supply, sanitation, and hygiene resources (Uprety et al., in press). There was a 80% increase in communicable waterborne infections in the first 6 months of 2015, including the 2 months after the April earthquake, as compared to years 2013–2014 combined [Department of Health Services (DOHS) of Nepal, 2016]. There are only a few studies examining the microbial community in water in Nepal, and these studies show the presence of multiple pathogens and multi-drug resistance species of bacteria (Pokhrel and Viraraghavan, 2004; Tanaka et al., 2012). Waterborne infectious disease outbreaks are a result of many factors, including person-to-person transmission, food contamination, poor sanitation, and water contamination through fecal-oral route (Yan and Sadowsky, 2007; Grandesso et al., 2014; Ashbolt, 2015). More recently, it has been appreciated that environmental conditions that favor an increased load of pathogens in water also are crucial factors contributing to outbreaks of waterborne diseases, as was the case for Haiti in 2010 (Lobitz et al., 2000; Jutla et al., 2013). However, it is not known how the dynamics of water microbial communities change after a catastrophic earthquake that destroys sanitation and water infrastructure.

To fill a knowledge gap regarding changes in environmental microbial communities' due to the 2015 earthquake, we collected source drinking water samples in Kathmandu and Jhapa in Nepal, two regions that were affected and unaffected by earthquakes, respectively. We performed 16S rRNA gene sequencing on three sets of water samples. The first set of samples were collected 11 months prior to the earthquake, and the remaining sample sets were collected 1–3 months and then 8 months after the earthquake. Microbial source tracking was also performed using human and cow specific markers to better understand the change in sanitation practices along with the change in microbial community. To our knowledge, this is the first study that probes water microbiome dynamics with respect to earthquakes.

## Materials and methods

### Study site

**Figure 1:**
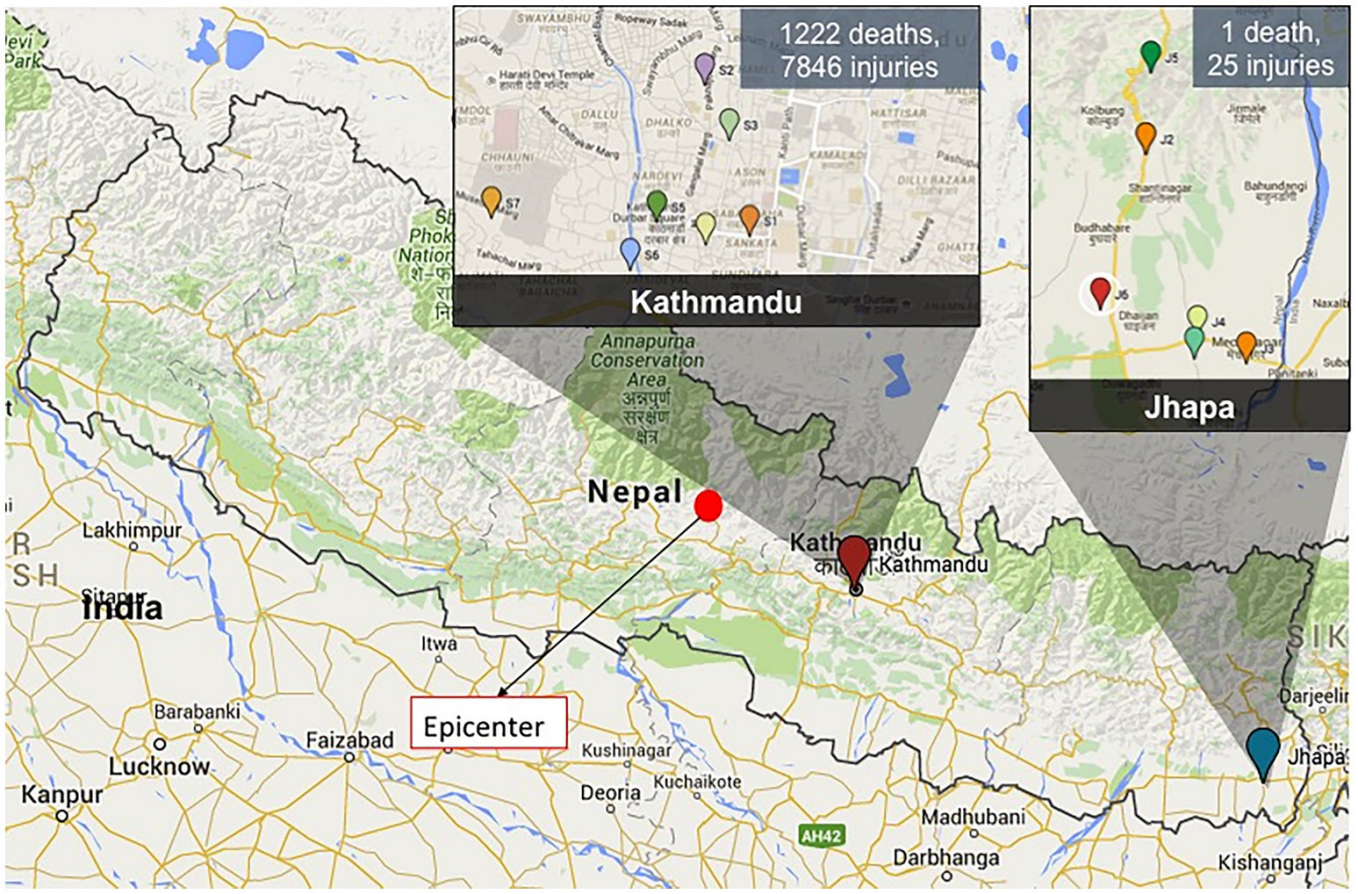
A map showing the epicenter of the 2015 Nepal earthquake (filled red circle) and the sampling locations in Kathmandu and Jhapa. The magnitude of damage in two sampling locations is shown as well.

Water samples were collected at seven schools in Kathmandu (S1–S7) and four households in Jhapa (J2–J5) at four different time points occurring from May 2014 (Figure 1, Table 1). This is referred to as Batch 1. All schools in Kathmandu were selected because these schools' water sources historically contained high concentrations of fecal and total coliform counts. All schools (S1–S7) are in central Kathmandu in an urbanized area with high population density, and groundwater is the water source for all schools. Apart from S2, which has unprotected bore holes, all sites have unprotected dug wells. Students used the school's water source and water brought from home as drinking water. The seven schools are government-owned and accommodated children mostly from lower-middle class families.

**Table 1:** Sampling location with GPS coordinates, water source type, level of earthquake damage and sampling Batches for each site.

**Site**	**Location**	**GPS coordinates**	**Water source type**	**Location type**	**Earthquake damage**	**Sampling batches^a^**
S1	Kathmandu	N27°42′44″ E 85°18′37″	Dug Shallow Well	School	High	Batch 1, 2, and 3
S2	Kathmandu	N27°42′53″ E 85^*^18′27″	Borehole Deep Well	School	High	Batch 1, 2, and 3
S3	Kathmandu	N 27°42′38″ E 85°18′37″	Dug Shallow Well	School	Damaged	Batch 1
S4	Kathmandu	N 27°42′10″ E 85°18′30″	Dug Shallow Well	School	Damaged	Batch 1
S5	Kathmandu	N 27°42′16″ E 85°18′15″	Dug Shallow Well	School	High	Batch 1, 2, and 3
S6	Kathmandu	N 27°42′03″ E 85°18′07″	Dug Shallow Well	School	High	Batch 1, 2, and 3
S7	Kathmandu	N 27°42′17″ E 85°17′25″	Dug Shallow Well	School	High	Batch 1, 2, and 3
J2	Jhapa	N 26°46′19″ E 88°04′13″	Surface water	Household	Low	Batch 1 and 2
J3	Jhapa	N 26°46′19″ E 88°04′19″	Surface water	Household	Low	Batch 1 and 2
J4	Jhapa	N 26°39′44″ E 88°06′20″	Surface water	Household	Low	Batch 1 and 2
J5	Jhapa	N 26°42″44″ E 88°05′21″	Surface water	Household	Low	Batch 1 and 2

a *Batch 1 = May-August 2014; Batch 2 = May-August 2015; Batch 3 = December 2015 water samples*.

*High earthquake damage indicates severe damage in infrastructure; low earthquake damage indicates minimum to no damage in infrastructure and damaged indicates the sampling site was inaccessible after the earthquake*.

Households in Jhapa (J2–J5) were also selected because their water source historically contained abundant fecal and total coliforms. In Jhapa, the drinking water source is river water, which is collected in a reservoir and piped to individual houses. Water samples (J2–J5) were taken from the household tanks piped from the river. Most of the families in the selected households relied on subsistence farming and had little or no formal education. However, due to various Water, Sanitation and Hygiene (WASH) campaigns conducted in the area, community members have been informed about basic sanitation and safe water practices.

After several earthquakes in April and May of 2015 (epicenters marked in Figure 1), additional water samples were collected at four time points from May to August 2015 (Batch 2) from the same locations in Kathmandu and Jhapa with some exceptions (Figure 1). The earthquake heavily affected Kathmandu, and as a result, two schools (S3 and S4) were not accessible for the second round of sampling. All sites in Jhapa were sampled during this same time frame because there were very limited effects of the earthquake on Jhapa compared to Kathmandu.

In December 2015, an additional water sample was collected again from the same sampling sites in Kathmandu (Figure 1). However, no samples were collected in Jhapa in December 2015 due to an ongoing fuel crisis in Nepal at the time that prohibited travel.

### Sampling protocol

Two-liter water samples were collected directly from faucet at each sampling site in sterile Whirl-pak® sampling bags (Nasco, WI) and were processed within 24 h of collection. Careful precautionary steps were taken during sampling to avoid cross contamination including changing of gloves between each sampling and sterilizing the cooler before and after each sampling. Samples of Kathmandu and Jhapa were collected and processed successively, so that there were no chances of cross contamination between the samples from two sites.

Samples in Kathmandu were collected directly from the well using the bucket provided and the samples in Jhapa were collected after a quick flush of 30 s. Samples were treated with 2.5 M MgCl_2_•6H_2_O (Sigma-Aldrich, St. Louis. MO) for 30 min to coagulate the microorganisms (Mattioli et al., 2013; Sadik et al., 2017). Next, coagulated water samples were vacuum-filtered through a 0.45 μm sterile cellulose acetate filter (GVS Maine, Sanford, ME) placed in 47 mm filtration funnel (Pall Corporation, New York, NY) for samples taken in 2014 (referred to as Batch 1 samples). However, this process clogged the 0.45 μm cellulose acetate filters very rapidly and was not feasible for practice on-site after the earthquake. Hence, water samples collected 1 year and year and half post-earthquake (Batch 2 and Batch 3, respectively) were vacuum-filtered through a 1.6 μm glass fiber membrane (Fisher Scientific, Hamptoon, NH) followed by a 0.45 μm cellulose acetate membrane after coagulation in a solution containing 25 mM magnesium chloride. During sample processing, the filtration unit was sterilized between each sample using disposable chlorine and ethanol wipes to avoid contamination. All working surface was thoroughly wiped with chlorine and ethanol wipe frequently during the sample processing. Sample membrane were then treated with RNAlater (Qiagen, Helden, Germany) and were stored in sterile Whirlpak® bags at −20°C until transport to University of Illinois at Urbana Champaign (UIUC). At UIUC, samples were stored at −80°C until extraction.

### DNA extraction

Total DNA for the biomass retained on 0.45 μm membrane was extracted using the MoBio PowerWater RNA Isolation Kit (Yu and Morrison, 2004), removing the DNase step to ensure the collection of both DNA and RNA. RNA was then removed by treating the extracted nucleic acids with RNase, followed by standard sodium acetate–ethanol precipitation to concentrate the DNA. Total DNA for the biomass retained on 1.6 μm membrane was extracted using the MPI FastDNA Kit for Soil Extraction (Smith et al., 2012) with minor modifications. The minor modification includes the repeat of ethanol precipitation four times instead of once as recommended in the manufacture's protocol. Extra ethanol precipitation was needed to remove the high concentration of salts present in the RNAlater used to stabilize RNA during sample storage and transportation. For Batch 1 samples, DNA from the 0.45 μm filter membrane was used for analysis of microbial community. For Batch 2 and Batch 3 samples, combined DNA in equal volumes from both 1.6 and 0.45 μm filters was used for microbial community analysis to best approximate the total biomass that would have been captured by the coagulation–filtration protocol used for Batch 1 samples. All nucleic acid extractions of the samples were carried out in a sterile hood at the UIUC and all recommended precautionary steps were taken during extraction to avoid contamination. The only bacteria being grown in the lab at the time was *Legionella*, and since *Legionella* was not detected in any of the samples, we are confident that the steps taken to avoid contamination were successful.

### PCR-based fecal source tracking

Microbial source tracking was performed using three primer pairs that target human-associated *Bacteroides uniformis, Bacteroides fragilis*, and *Bacteroides vulgatus* and a primer pair that targets cow-specific uncultivated *Bacteroidales*. Gene inserts were obtained from *B. vulgatus* BCRC12903, *B. uniformis* JCM5828, *B. fragilis* BCRC10619, and from a cow-specific uncultivated *Bacteroidales* clone obtained from an earlier study (Hong et al., 2009). qPCR standards were prepared by first cloning the gene inserts into pCR4 TOPO vector (Invitrogen, Carlsbad, CA, USA). Plasmid DNA was extracted using PureYield™ Plasmid Miniprep System (Promega, Madison, WI, USA). The extracted plasmids were sequenced to verify the oligonucleotide sequences of gene inserts and quantified. PCR amplifications were performed with each plasmid to obtain standard curves. These experiments were performed in triplicate, while PCR amplification of experimental samples or negative controls was run in duplicates. Each PCR reaction volume of 20 μL contained 10 μL of FAST SYBR Green master mix, 0.4 μL of each primer (10 μM), 1 μL of DNA template (10–400 ng), and 8.2 μL molecular biology grade water. The Applied Biosystems 7900 HT Fast protocol was used for thermal cycling. The protocol includes 40 cycles of 1 s denaturation at 95°C and 60 s of annealing and extension. Dissociation curve analysis was included to detect non-specific amplification. The qPCR assays used in this study are the same as that previously reported (Zhang et al., 2014). The sensitivity and specificity assessment of these assays were evaluated and the LOD for the human-associated Bacteriodales primer assays are 1.3 × 10^3^, 1.9 × 10^3^ and 1.7 × 10^3^ copies/ng genomic DNA for Bvg, Bfrg and Bufm primer pairs respectively. Also, the LOD for the cow-specific primer assay was determined to be 4.7 × 10^2^ copies/ng genomic DNA. The LOQ of human-associated Bacteriodales primer assays were 1.3 × 10^9^, 1.9 × 10^8^ and 1.7 × 10^8^ copies/ng DNA for Bvg, Bfrg and Bufm primer pairs respectively and that for cow-specific Bacteriodales was 4.7 × 10^7^ copies/ng DNA.

### 16S rRNA gene-based amplicon sequencing and data analysis and statistics

Illumina MiSeq amplicon sequencing was performed for all the samples to provide information on the microbial community. To prepare the 16S rRNA gene amplicon libraries, 515F (5′-Illumina overhang-GTGYCAGCMGCCGCGGTAA-3′) and 907R (5′-Illumina overhang-CCCCGYCAATTCMTTTRAGT-3′) primers were modified to encode the overhang adaptor sequences, and used to amplify the 16S rRNA genes. The thermal cycling program included an initial denaturation stage at 95°C for 3 min, followed by 25 cycles of denaturation at 95°C for 30 s, annealing at 55°C for 30 s, and extension at 72°C for 30 s, followed by a final extension period at 72°C for 5 min. PCR amplicons were purified by AMPure XP beads (Beckman Coulter, CA, USA) prior to the index PCR assay. Nextera XT Index (Illumina, CA, USA) was incorporated into each of the individual samples during PCR. The thermal cycling program included denaturation stage at 95°C for 3 min, followed by eight cycles of denaturation at 95°C for 30 s, annealing at 55°C for 30 s and extension at 72°C for 30 s, followed by a final extension period at 72°C for 5 min. The final indexed PCR amplicons were again purified by AMPure XP beads, and nucleic acid concentrations were quantified using Invitrogen Qubit® 2.0 fluorometer. The controls for all PCR reactions were negative for amplification. Purified amplicons were submitted to KAUST Genomics Core lab for unidirectional sequencing read on an Illumina MiSeq platform. The sequences are deposited in the European Nucleotide Archive (ENA) under accession number PRJEB14325.

Raw sequences were first trimmed to remove the primers, barcodes, and adaptor sequences. Trimmed sequences that were <300 nt in length and with Phred score <20 were removed. Chimeras were identified using UCHIME (Edgar et al., 2011) by referencing to a core set that was downloaded from Greengenes (i.e., gold strains gg16—aligned.fasta, last modified on 19 March 2011). Chimeras were then removed from future analyses. The relative abundances of the bacterial and archaeal genera were then calculated, collated, and square-root transformed. The transformed data sets were computed for their Bray–Curtis similarities and represented graphically for spatial distribution and vector analysis in a non-metric multidimensional scaling (MDS) plot using Primer-E version 7.

Finally, two-way ANOVA test to analyze the statistical significance was tested for samples collected in several time periods for both Kathmandu and Jhapa samples. Samples were tested for May 2015–May 2015 samples, May 2014–July 2015 samples, and May–August and December 2015 samples. Significant change between two sampling periods were considered for *p* < 0.05. This statistical comparison suggested if the change in microbial communities were because of natural or seasonal variation or because of the earthquake.

## Results and discussion

Although the incidence of waterborne diseases usually increases dramatically after major natural disasters (Ivers and Ryan, 2006; Watson et al., 2007), there is very limited research on the direct impact on the changes in microbial communities of water and the potential impact of these changes on public health arising from earthquakes. Instead of analyzing the changes in the microbial community of water longitudinally to determine the direct impact of an earthquake (as we did here), most studies tend to examine the indirect impact at a given time due to earthquakes or earthquake-triggered tsunamis. Metagenomic analysis of soil microbial communities after the 2011 earthquake and tsunami in Japan revealed the loss of siderophore-synthesis genes from *Arthrobacter* strains, an over-representation of denitrification related genera of microbes, and the presence of pathogenic bacteria (Hiraoka et al., 2016). Similarly, a soil microbial ecology study conducted 7 years after the tsunami in the Phang Nga province in Thailand revealed the presence of more *Bacteriodes* and other pathogenic microbes as compared sites that were not affected by the tsunami (Somboonna et al., 2014). In instances where studies examined the anthropogenic impact on water sources due to earthquake damage, these studies typically examined samples collected during one sampling event after an earthquake. For example, an increase in the amount of pathogenic bacteria were present in water samples collected from earthquake-affected area in Pakistan as compared to the areas that were not affected by earthquake (Rasheed et al., 2009). Even though these studies provide some insight about disturbance in the microbial communities after an extreme natural event, the emphasis is largely on the detection of fecal indicators and pathogenic microorganisms at one time point. There still exists a knowledge gap for understanding the dynamics of microbial communities in response to natural disasters and this study begins to fill this gap. Our strategy to fill this gap was to analyze and compare changes in the microbial community of water longitudinally, both before and after events like monsoons and earthquakes.

### Characterization of microbial communities of water prior to the 2015 earthquake

**Figure 2:**
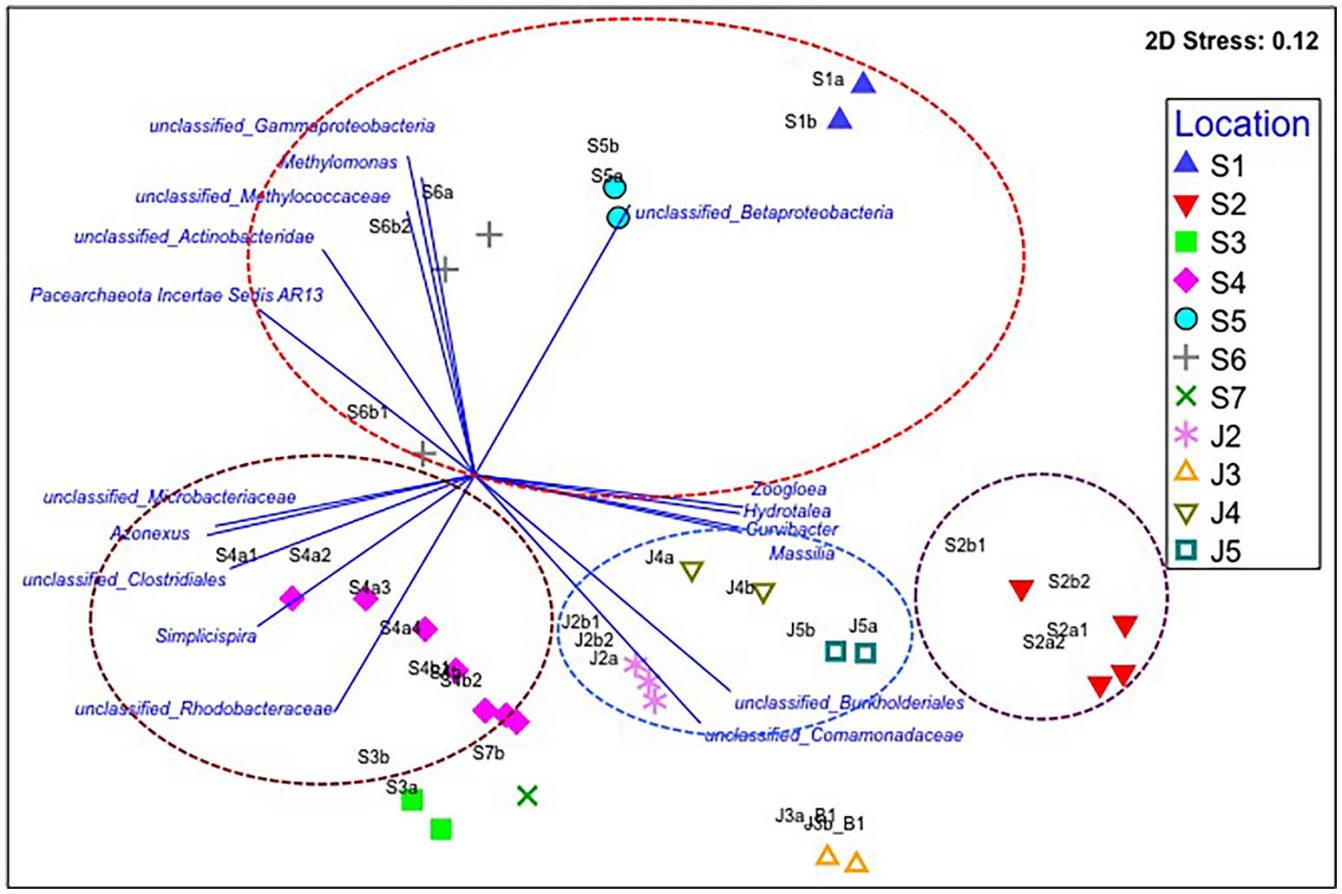
Non-metric multidimensional scaling (NMDS) plot for the averaged microbial communities in each Kathmandu (S1–S7) and Jhapa (J2–J5) water sample that was taken in (Batch 1). Vector-based analysis (blue lines and text) overlay the bacterial population that showed significant correlation with the clustering patterns. The letters a, b, c, d (e.g., S2a, S2b, S2c, S2d) represent different filter membranes used for each sample collected. More filter membranes were used at some sites (S2) compared to other sites (J3) because water turbidity was higher. Similar bacterial populations are indicated by circles of different colors.

We first determined microbial communities in water samples that were taken from Kathmandu and Jhapa in May 2014, a time prior to the earthquake, by using 16S rRNA sequencing. The relative abundance of known bacterial genera and unclassified bacterial groups in each water sample was compared to other samples using their Bray–Curtis similarities (Figure 2). These data revealed that the microbial communities of all four Jhapa water sources (J2–J5) shared 55% similarity and formed one cluster. We analyzed the bacterial communities using a non-metric multidimensional scaling plot coupled with vector-based analysis to confirm data from the Bray–Curtis similarities (Figure 2). We observed four bacterial populations that were prevalent in all four samples from Jhapa (Figure 2). For example, members of the order *Burkholderiales* accounted for 13, 25, 30, and 47% of total microbial community for samples J2, J3, J4, and J5, respectively (Figure 2). Members of family *Comamonadaceae* accounted from between 5 and 27% of the bacterial population in the water samples from Jhapa (Figure 2). The remaining two dominant bacterial population present in all Jhapa samples were members of Moraxellaceae family (3–18%) and *Flavobacterium* genus (1–7%) (data not shown in the plot).

In contrast to the water samples from Jhapa, the seven water samples collected in Kathmandu (S1–S7) clustered into three different groups when using Bray–Curtis similarities (Figure 2). Samples S1, S5, and S6 were clustered in one group and samples S3, S4, and S7 were clustered in another group (Figure 2). Sample S2 was in its own cluster. Bray–Curtis analyses also revealed that samples S3, S4, and S7 shared 51% similarity. Each of these three samples possessed members from the genus *Flavobacterium* and *Polynucleobacter*, which contributed on average to 18–8% of microbial community, respectively. However, each sample also possessed unique bacterial populations, which is why the Bray–Curtis score was not higher. For example, in sample S3, members of the *Comamondaceae* family (8.5% of the total microbial population) predominated, followed by members of the order *Burkholderiales* (6% of the total microbial community). In contrast, the predominant bacterial genera present in S4 were *Flavobacterium* and *Polynucleobacter* (26 and 11% of the population, respectively). In sample S7, family *Planctomycetaceae* and members of family *Comamonadaceae* were predominant, accounting for 18 and 13% of the total microbial community. Thus, although samples S3, S4, and S7 shared some bacterial members, the predominance of different bacterial orders, families, and genera in each community have less similarity as compared to Jhapa samples. When examining similarities in between water samples taken in Kathmandu, Bray–Curtis analyses revealed that samples S1, S5, and S6 clustered together with 43% similarity. In these samples, members of Gammaproteobacteria and Betaproteobacteria are the most abundant (23 and 20%, respectively). In contrast, populations of Gammaproteobacteria and Betaproteobacteria were 8 and 7% abundant, respectively, in S6. This is one reason why there was a decreased percentage similarity in this cluster.

Sample S2 shared only 38% similarity to all other Kathmandu samples (Figure 2), indicating a major difference in microbial communities between S2 and other Kathmandu sites. This low similarity was because members of the order *Burkholderiales* and the family *Comamonadaceae*, which were not predominant in other Kathmandu samples, accounted for 60% of the microbial community in total for S2 samples. S2 also possessed bacterial genera like *Azospira* (4%) and *Zoogloea* (3%), genera that were absent in other Kathmandu water samples.

It was also observed that there were differences in the abundance of bacterial or archaeal genera routinely known to be important for geobiochemical characteristics of water in water collected from Jhapa vs. Kathmandu (Azam and Smith, 1991). Water samples from both locations possessed *Nitrospira* and *Nitrososphaera* (Table 2). However, genera like *Nitrosopumilus, Methylobacter, Methylomonas*, and *Desulfovibrio* were only detectable in water samples from Kathmandu (Table 2). Together these data indicate that drinking water microbiomes in Jhapa are (i) more similar to each other than those in Kathmandu, and (ii) distinct from those in Kathmandu.

**Table 2:** The average relative abundance of genera associated with geochemical characteristics of water in Kathmandu (S1–S7) and Jhapa (J2–J5).

**Bacterial/Archaeal genera**	**Type of Bacteria/Archaea^a^**	**Average Kathmandu**	**Average Jhapa^b^**
*Nitrospira*	NOB	0.182%	0.086%
*Nitrososphaera*	AOA	0.007%	0.003%
*Nitrosopumilus*	AOA	0.045%	ND
*Methylobacter*	MOB	0.005%	ND
*Methylomonas*	MOB	0.025%	ND
*Desulfovibrio*	SRB	0.004%	ND

*Samples were collected from May to August 2014*.

a *NOB, Nitrite Oxidizing Bacteria; AOA, Ammonia Oxidizing Archaea; MOB, Methane Oxidizing Bacteria; SRB, Sulfate Reducing Bacteria*.

b *ND, not detected*.

The differences in microbial communities in samples from Jhapa vs. Kathmandu pre-earthquake are likely reflective of the different water sources used by each community, different climate conditions, and different human activities. Source water in Kathmandu is from a single aquifer (Khatiwada et al., 2002), which is then accessed by a deep or shallow well. In contrast, households in Jhapa rely on river water that is stored and distributed through a shared reservoir. Surface water and groundwater environments have distinct indigenous microbial communities (Griebler and Lueders, 2009).

We also observed distinct microbial communities in water samples taken in Kathmandu (Figure 2). All sampling sites in Kathmandu were shallow/dug wells, except S2 which is a deep borehole (24 m) well. Thus, the microbial communities from the S2 samples were distinct from other urban samples (Figure 2). Furthermore, among the samples from the shallow wells, the formation of different clusters by S1, S5, and S6 vs. S3, S4, and S7, could be due to differences in sanitation practices at these locations. Notably, S3 and S7 samples were from wells that are only ~10 and ~4 m, respectively, from pit latrines, where there could be seepage of bacteria from human waste into drinking water sources.

### Comparison of microbial communities from samples collected in May 2014 vs. May 2015

Only Jhapa samples J3 and J4, and Kathmandu samples S2 and S5 were reliably accessible throughout all three sampling periods (as some schools were destroyed after May 2015 earthquake in Kathmandu). For these reasons, we focused on these samples for the analyses shown in Table 3. Namely, we examined the microbial communities from the same water sources 12 months later (in May 2015; Figure 1) to ask if bacterial communities changed longitudinally. We performed two-way ANOVA analyses to determine statistical significance for all samples collected in May 2014 and May 2015 in both Kathmandu and Jhapa (Table 4, columns 1 and 2). Results showed that that difference was not significant between Kathmandu samples collected in May 2014 and May 2015 (*p* > 0.05) except for *Methylomonas* (Table 4). For Jhapa samples, there were no statistically significant changes (*p* > 0.05, Table 4, column 2) for all selected genera except *Nitrospira, Legionella*, and *Aeromonas*.

**Table 3:** Fold- difference in relative abundance of bacterial genera in Kathmandu and Jhapa water samples collected in May 2014 vs. May 2015 (dry season), or May 2015 vs. July 2015 (dry to wet season transition).

**Bacterial genera**	**Fold change in S2 (dry season)**	**Fold change in S5 (dry season)**	**Fold change in J3 (dry season)**	**Fold change in J4 (dry season)**	**Fold change in S2 (dry to wet season)**	**Fold change in S5 (dry to wet season)**	**Fold change in J3 (dry to wet season)**	**Fold change in J4 (dry to wet season)**
*Methylobacter*	N/A (ND to ND)	0.45 (0.028 to 0.014%)	N/A (ND to ND)	N/A (ND to ND)	N/A (ND to 0.50%)	94.8 (0.03 to 2.7%)	N/A (ND to ND)	N/A (ND to ND)
*Desulfovibrio*	593.47 (0.008 to 4.71%)	N/A (ND to ND)	N/A (ND to ND)	N/A (ND to ND)	42.4 (0.008 to 0.337%)	N/A (ND to 0.016%)	N/A (ND to ND)	N/A (ND to ND)
*Nitrospira*	N/A (ND to ND)	4.64 (0.014 to 0.065%)	0.48 (0.078 to 0.037%)	N/A (ND to ND)	N/A (ND to 0.027%)	2.4 (0.0139 to 0.033%)	4.50 (0.037 to 0.165%)	N/A (ND to 0.26%)
*Methylomonas*	N/A (ND to ND)	2.13 (0.042 to 0.091%)	N/A (ND to ND)	N/A (ND to ND)	N/A (ND to 0.08%)	3.14 (0.042 to 0.133%)	N/A (ND to ND)	N/A (ND to ND)
*Acinetobacter*	1.71 (0.063 to 0.180%)	N/A (ND to 1.772%)	4.25 (0.131 to 0.560%)	0.66 (0.174 to 0.116%)	2.6 (0.06 to 0.162%)	N/A (ND to 0.43%)	0.07 (4.717 to 0.337%)	121.04 (0.12 to 13.97%)
*Aeromonas*	N/A (0.007% to ND)	N/A (ND to 0.71%)	N/A (0.020% to ND)	N/A (ND to ND)	N/A (0.007% to ND)	N/A (ND to 0.433%)	N/A (ND to 0.147%)	N/A (ND to 0.03%)
*Legionella*	N/A (ND to ND)	N/A (ND to ND)	1.03 (0.012 to 0.013%)	N/A (ND to ND)	N/A (ND to 0.013%)	N/A (ND to ND)	2.35 (0.012 to 0.03%)	N/A (ND to 0.01%)

*N/A, not applicable, fold change cannot be calculated due to the absence of a genus in one of the samples. ND, not detected*.

**Table 4:** Statistical significance for samples collected in different time periods.

**Bacterial genera**	***p*-value May 2014–May 2015 Kathmandu**	***p*-value May 2014–May 2015 Jhapa**	***p*-value May 2014–July 2015 Kathmandu**	***p*-value May 2014–July 2015 Jhapa**	***p*-value May-August and December 2015 Kathmandu**
*Nitrospira*	0.07	0.01	0.90	0.09	0.04
*Methylobacter*	0.08	N/A	0.29	N/A	0.08
*Desulfovibrio*	0.27	0.17	0.41	0.18	0.28
*Methylomonas*	0.03	N/A	0.42	N/A	0.04
*Legionella*	0.79	0.01	0.35	0.54	0.02
*Aeromonas*	0.14	0.05	0.76	0.07	0.02
*Acinetobacter*	0.11	0.10	0.48	0.27	0.95

*N/A, not applicable, p-value cannot be calculated due to the absence of a genus in one of the samples*.

In addition to examining bacteria associated with biostability of water, shown in Table 2, we also investigated if *Legionella, Aeromonas*, and *Acinetobacter* were present because they are opportunistic pathogens commonly found in water (Madigan et al., 2008). For Jhapa water samples, there was an increase in population for only one of the three bacterial genera that cause opportunistic infections (4.25-fold increase in *Acinetobacter* populations; Table 3, column 3). Also, there was no dramatic change in the abundance of the bacterial genera associated with geobiochemical characteristics of water (Table 3, columns 3 and 4). In the Kathmandu water samples, the relative abundance of several bacterial populations increased between May 2014 and May 2015 (Table 3, columns 1 and 2). The largest increase was that of *Desulfovibrio* spp., for which there was a 593-fold increase in S2 comparing May 2014 to May 2015 samples. For S5, a 4.64-fold increase in *Nitrospira* spp. and a 2.13-fold increase in *Methylomonas* spp. were observed between 2014 and 2015 (Table 3). The absence of geobiochemically-relevant bacteria, *Methylobacter, Desulfovibrio*, and *Methylomonas*, in Jhapa water samples collected almost a year apart (Table 3) suggested negligible methyl-oxidation and sulfate-reduction in water from Jhapa.

**Figure 3:**
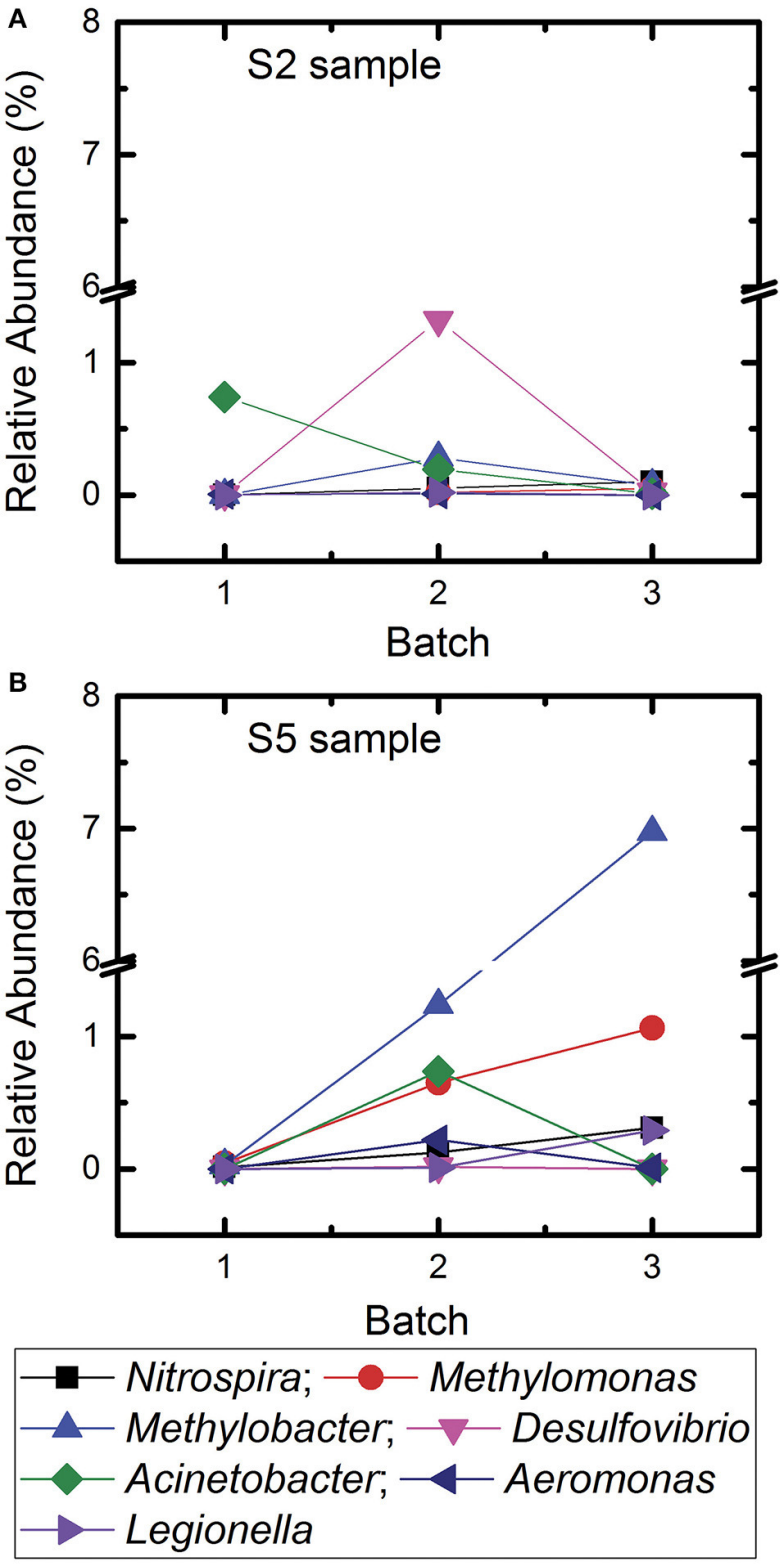
Change of relative abundances of different bacterial genera associated with biogeochemical characteristics of water and genera associated with opportunistic pathogens in samples S2 and S5 for Batch 1, Batch 2, and Batch 3. **(A)** Sample site S2 and **(B)** sample site S5.

In contrast, we observed an increase in the relative abundance of bacterial genera that are responsible for maintaining geobiochemical characteristics (e.g., *Nitrospira, Desulfovibrio, Methylomonas*) of water post-earthquake for S2 and S5 in Kathmandu (Figure 3, Table 3). We suggest that the changes in these populations were a result of the April 2015 earthquake. Indeed, others have reported similar bacterial populations in water quality after a natural disaster (Ivers and Ryan, 2006; Rasheed et al., 2009; Hiraoka et al., 2016). Although not all locations were accessible for sampling, the analysis for all collected samples may not reflect the changes in microbiome before and after the earthquake, based on the available data we conclude that, in general, bacterial populations changed longitudinally to a greater degree in Kathmandu samples vs. Jhapa samples.

### Seasonal changes in microbial community collected in May 2015 (dry season) and July 2015 (monsoon season)

We next compared the microbial communities of water samples collected in dry vs. monsoon seasons in 2015 to identify (i) the natural variation in water microbiomes collected from Jhapa, and (ii) the impact of the 2015 earthquake in water microbiomes from Kathmandu. We first conducted a two-way ANOVA analysis of data from May 2015 and July 2015 samples taken from both locations. There were no statistically significant changes in bacterial genera examined in May 2014–July 2015 for both Kathmandu and Jhapa samples (Table 4). Note again that only two sites from Jhapa (J3 and J4) and two sites from Kathmandu (S2 and S5) were selected for further analysis for bacteria associated with opportunistic infections. For the Jhapa samples J3 and J4, where the effect of the 2015 earthquake was minimal, only *Nitrospira* and *Acinetobacter* populations increased (Table 3). During this same transition period in Kathmandu, *Methylobacter* populations showed the largest change in relative abundance, for which there was a 94.8-fold increase in sample S5. *Desulfovibiro* populations increased by 42.4-fold in sample S2. Smaller increases in *Nitrospira* and *Aceintobacter* populations were observed in samples S5 and S2, respectively. Thus, the changes in populations of bacterial genera associated with geobiochemical characteristics of water were more pronounced in Kathmandu vs. Jhapa samples. In addition, changes in microbial communities were more pronounced when comparing pre- vs. post-earthquake to dry vs. wet season communities.

### Microbial community dynamics in Kathmandu 6 months post-earthquake

In December 2015, samples were collected from S2 and S5 locations in Kathmandu to determine if the microbial communities approximated toward the relative abundances of microbes detected in 2014 samples (Figure 3). Jhapa water samples were not collected in December 2015 because results from Table 3 suggested that there were minimal changes in microbial communities over time in Jhapa.

In the S2 sample, *Methylobacter* was not detected in 2014 samples. By May–August 2015, *Methylobacter* contributed to 0.3% of total microbial community. By December 2015, it contributed to 0.07%. Similarly, *Desulfovibrio* spp. contributed to 0.006% of the microbial community in 2014 samples. This contribution increased to 1.32% in May–August 2015 samples, and then decreased to 0.025% by December 2015. Thus, it appeared that the population of *Desulfovibrio* spp. was returning to levels observed pre-earthquake. The trend was also observed in bacteria that are opportunistic pathogens. In the S5 sample, *Acinetobacter* was not detected in 2014 samples but contributed to 0.73% of total microbial community in the samples collected 3 months after the earthquake. However, *Acinetobacter* was not detected in December 2015. Similarly, *Aeromonas* was not detected in 2014 was detected in May–August 2015 (0.21%) but decreased to 0.009% in December 2015, returning closer to 2014 samples.

In addition, there was an increase in relative abundance of other bacterial genera throughout the sampling periods. For example, the relative abundance of *Methylomonas* in the S5 samples increased from 0.04% to 0.65% to 1.063% in Batch 1, Batch 2, and Batch 3, respectively. In addition, *Methylobacter* increased from 0.028 to 1.23% over time. In summary, the microbial communities in Kathmandu water shifted after the earthquake. In some cases, populations of waterborne bacteria returned to the levels observed in 2014.

The earthquake also changed in human activities and behaviors, changes will also alter microbial communities in Kathmandu. For example, the creation of and changes in population sizes for temporary settlements (which were in response to the earthquake) may affect water microbiomes. Indeed, human settlement related to mining in Brazil drives the abundance of nitrifying bacteria and archaea (Reis et al., 2015). Similarly, human activities cause disturbances of methane-oxidizing bacteria like *Methylomonas* (Holmes et al., 1999) and also ammonia oxidizers (Ying et al., 2010). Thus, we speculated that the increase in geobiochemically relevant bacterial genera in Kathmandu samples may be related to human activities.

Two-way ANVOA analyses showed no statistically significant differences between *Acinetobacter, Methylobacter*, and *Desulfovirbiro* (*p* > 0.05) in samples collected between May–July 2015 and December 2015. However, there was a statistically significant change (*p* < 0.05) in *Nitrospira, Methylomonas, Legionella*, and *Aeromonas* populations at that same time (Table 4). When examining samples S2 and S5, we observed a dramatic increase in geobiochemically relevant bacterial genera (e.g., *Desulfovibrio* and *Nitrospira*) in S2 and S5 samples soon after the 2015 earthquake. These bacterial populations decreased to pre-earthquake levels by December 2015 (Figure 3). Similar trends were observed for *Acinetobacter* and *Aeromonas* in these same water samples (Figure 3). This observation indicates that, despite the shift in the microbial community that occurred immediately after the earthquake, the microbial community was returning to a profile similar to those observed prior to the earthquake. We speculate that this return was due mostly to the closing of temporary settlements, which would decrease unsafe sanitation practices and the nitrate and ammonia load in water, providing an environment that is conducive to proliferation of indigenous microbiota.

### PCR-based fecal source tracking

Host-associated Bacteroidales is used as bacterial indicator to identify an originating source of fecal contamination (Jenkins et al., 2009). Using this system, a sample is considered positive for human fecal contamination when two or more human-associated *Bacteroides* spp. are present in a sample (Hong et al., 2009). We used this approach as an additional method to indicate water quality and sanitation conditions for samples S2 and S5 longitudinally. Results are shown in Table 5. Samples from S2 were negative for all three human-associated *Bacteroides* spp. markers examined both pre- and post-earthquake. However, these same samples were positive for a cow-specific *Bacteroidales* (Hong et al., 2009) marker only at one time point (immediately after the earthquake), implying the presence of cow feces near this sampling site. For site S5, human fecal contamination was detected at all-time points (Table 5). However, cow-specific *Bacteroidales* markers were not detected in any of the S5 samples. It is to be noted that S2 water comes from a deep well, whereas S5 water comes from a shallow well. Moreover, these data suggest that there was more fecal contamination after the earthquake in Kathmandu.

**Table 5:** Presence or absence of human-associated *Bacteroides* spp. and cow-specific *Bacteroidales*.

	**Sample**	**Positive for 2 or more human markers**	**Positive for cow marker**
Batch 1 (May 2014)	S2	−	−
	S5	+	−
Batch 2 (July 2015)	S2	−	+
	S5	+	−
Batch 3 (Dec. 2015)	S2	−	−
	S5	+	−

The increase in human-specific *Bacteroides* and/or cow-specific *Bacteroidales* detected in the water samples collected post-earthquake indicated compromised sanitation practices. Sites S2 and S5 were being used as temporary camps for the victims of the earthquake. Open defecation due to the lack of toilets near the camps is expected to introduce fecal contamination to the water sources. One expectation is that the human and animal-associated Bacteroides will decrease over time, as the people of Kathmandu rebuild infrastructure.

### Study limitation

This study presents new knowledge on the dynamics of water microbiota after the Nepal 2015 earthquake and demonstrates the restoration of the water microbiome over time. There were limitations to this study. First, although 16S rRNA gene-based sequencing can mostly characterize bacterial genera, information related to viruses and eukaryotes including fungal and parasitic genera are not included. Second, 16S rRNA gene-based amplicon sequencing also does not provide information related to the functional genes, which play important roles in the overall nutrient and biogeochemical cycling and those related to virulence-associated genes. Third, although this study aims to assess the degree of perturbation as a function of time, sampling immediately after the earthquake and 8 months after the earthquake may not be enough to comprehensively characterize all important genera as restoration properties may differ among genera. Fourth limitation of this study is on the sequencing control. While we conducted the sample extraction to the best of our ability, complete avoidance of contamination was not confirmed. The sequencing control was done in accordance to the specifications suggested by Illumina for low diversity libraries such as amplicon libraries. Specifically, PhiX was added at 20% to provide a spike-in internal control to monitor sequencing quality based on cluster density, base alignment error rates. All samples were monitored based on these parameters and those sequencing libraries that do not meet the quality control are discarded. Results in this study are those that pass the sequencing control quality check. However, since PhiX was used as Illumina's internal sequence, sequencing negative controls with samples collected throughout the study is a more reliable way to check on contamination due to reagents and laboratory condition, as suggested in Salter et al. (2014). In our study, we did not observe a microbial population that occurred consistently throughout all samples to indicate background contamination. For future study, we recommend including sequencing of negative control throughout the sample extraction and preparation for sequencing. To overcome these limitations, future studies will use shotgun metagenomics sequencing of samples collected longitudinally to understand the overall microbial diversity, including viruses, rather than be limited to 16S, 18S, and 23S rRNA genes (Riesenfeld et al., 2004; Edwards and Rohwer, 2005; Tringe et al., 2005). In addition to 16S rRNA sequencing, viability assay, metagenomics, and metatranscriptomics will allow a more comprehensive understanding of the microbial communities and their functions. Future studies will also aim to increase the frequency of sampling post-earthquake to better understand the kinetics of restoration of a microbial community in the source water. Despite the limitations, the results of this study provide an improved understanding on the change in microbial communities of water under the influence of seasonal variation and a large-scale earthquake.

## Author contributions

SU: Field and lab work, manuscript writing and reviewing, data analysis. P-YH: Lab work, data analysis, manuscript writing and reviewing. NS: Field and lab work, manuscript writing and reviewing. BD: Manuscript writing and reviewing. RA: Field work, manuscript writing and reviewing. AJ, JS, and PD: Manuscript writing and reviewing, technical support. TN: Corresponding author, manuscript writing and reviewing, technical support, data analysis.

### Conflict of interest statement

The authors declare that the research was conducted in the absence of any commercial or financial relationships that could be construed as a potential conflict of interest.
